# Chemical and nutritional characteristics of *Cannabis sativa* L. co‐products

**DOI:** 10.1111/jpn.13557

**Published:** 2021-08-26

**Authors:** Alessandro Vastolo, Serena Calabrò, Severina Pacifico, Bossima Ivan Koura, Monica Isabella Cutrignelli

**Affiliations:** ^1^ Department of Veterinary Medicine and Animal Production University of Napoli, Federico II Napoli Italy; ^2^ Department of Environmental, Biological and Pharmaceutical Sciences and Technologies University of Campania Luigi, Vanvitelli, Caserta Italy; ^3^ Ecole de Gestion et d’Exploitation des Systèmes d’Elevage Universitè Nationale d’Agriculture Ketou Benin

**Keywords:** hemp, *in vitro* gas production, methane emission, ruminant

## Abstract

*Cannabis sativa* L. is an annual herbaceous plant. It was used for centuries to obtain different products. In the last century, hemp cultivation was forbidden due to the psychoactive effects of tetrahydrocannabinol acid (THCA). In the last years, new strains, characterized by high cannabidiolic acid (CBDA) and low THCA level, were developed renewing the interest in hemp cultivation to obtain food or to extract essential oils from flowers. All these processes produce many residues with different chemical–physical characteristics. In order to evaluate their potential use also in animal nutrition, some hemp co‐products were evaluated. Two different co‐products of seed processes (flour and oil) and two co‐products obtained trimming the flowers, differing in granulometry were used. The samples were analysed for chemical composition and evaluated *in vitro* using the gas production technique with buffaloes' ruminal *inoculum*. All hemp co‐products showed interesting nutritional characteristics, such as crude protein content always higher than 20% on a dry matter basis, and high neutral detergent fibre concentration partially lignified. The *in vitro* gas production parameters at 120 h of incubation showed quite low fermentability testified by the low organic matter degradability and cumulative gas volume (OMD from 28.09 to 45.64% and OMCV from 110 to 164 ml/g, respectively). Also, the methane produced after 24 h of incubation was particularly low (from 1.78 to 11.73 ml/g dOM). These results could be due to the high lipid and ash amounts or to the CBDA content that probably affected the CH_4_ formation processes. According to preliminary results obtained by this study, it is possible to hypothesize that these co‐products could be useful to mitigate the methane production into the rumen. Further studies are necessary in order to evaluate the correct inclusion into the diet for ruminants.

## 1 INTRODUCTION

Hemp is a dicotyledonous plant from the family of *Cannabaceae*, genus *Cannabis*, and is considered one of the oldest domesticated crops. For several years, this plant was used for different purposes, such as clothing and shoes, cordages, carpets and tarps, maritime ropes, sails and nets, and paper production (Crini et al., 2020). However, in the last century, hemp cultivation was decreased due to several reasons. First, the competition with other natural fibres such as cotton and jute for textile applications, and the intensive development of synthetic fibres. Moreover, *Cannabis sativa* L. was confused with *Cannabis indica* L., known for its high level of Δ^9^‐tetrahydrocannabinol acid (THCA), a chemical compound responsible for some psychoactive effects. This relation is, nevertheless, incorrect, indeed *Cannabis sativa* L. has <0.2–0.3% of TCHA (Milanovic et al., 2012; Żuk‐Gołaszewska & Gołaszewski, 2018). Moreover, cultivars of *Cannabis sativa* L. are rich in cannabidiolic acid (CBDA), the main phytocannabinoid of this plant particularly concentrated in its products (fibre and seeds) (Fiorini et al., 2019).

Nowadays, the renewal of hemp cultivation was increased throughout the world. In Europe, the cultivation areas for hemp increased over 50% (from 12,232 to 24,939 ha) from 2008 to 2018 (FAOSTAT, 2020). Indeed, hemp could be considered a valid alternative to the conventional crops produced in excess due to its limited environmental impact (less use of water and land exploitation). Hemp provides many agricultural benefits, such as weed control, pest and disease resistance, fast growth, capacity to remove significant quantities of heavy metals from the soil (bioremediation), and to provide high biomass production with low inputs. These characteristics make *C*. *sativa* an adaptable, affordable, and ecological crop (Ranelli & Venturi, 2004).

The cultivation and processing of hemp have many purposes, especially in food production to obtain many products, such as decorticated seeds, flour and oil. These products are considered sources of essential fatty acids and dietary fibre and are used as ingredients for several food preparations such as biscuits, bread, and protein powder. The seeds of *C*. *sativa* present on average 25–35% of lipids and 20–25% of proteins. The fatty acid profile of hemp seeds includes ω3 and ω6 essential fatty acids; in particular, the oil shows an abundance of α‐linolenic acid providing a ω6/ω3 ratio nearly to 2.5–3.0/1.0, within the range indicated as optimal for human dietary recommendations (Faugno et al., 2019). Additionally, hemp is also rich in natural antioxidants and other bioactive components (peptides, phenolic compounds, tocopherols, carotenoids and phytosterols) (Farinon et al., 2020).

During the flowers and seeds processing to obtain hemp prepared food various residues are produced. We have hypothesized that these co‐products could be used also for ruminant nutrition considering their nutritional properties. The study aimed to characterize hemp co‐products in terms of chemical composition and *in vitro* fermentation characteristics. The amounts of phytocannabinoids were also measured.

## 2 MATERIALS AND METHODS

### 2.1 Sampling

All samples were collected in a storage shed, sited in the province of Caserta (South Italy). Totally four different hemp co‐products were evaluated. All evaluated substrates derive from the same cultivar (Futura 75). This cultivar, particularly widespread in South Italy, is usually sowed, for seed production, in March–April and mechanically harvested in September–October. While the flowers were obtained from previous harvesting (July–September), immediately after, the apical portion of the plant was dried 21–23°C for 10 days, subsequently, the flowers were mechanically separated (trimmed) and a lot of biomass were discarded. Two co‐products were obtained after trimming the flowers and sieving at different granulometry (rough_FR and thin_FT). This biomass was partially chopped, and, for the evaluation, we have separated by sieving the chopped biomass into two portions, higher or lower than 4 mm. Considering one kilo of biomass 565 and 435 g of FR and FT were respectively obtained.

Two co‐products were obtained from the seed processing, which was made within 20 days from the harvesting. SF was the hemp oil meal obtained by cold mechanical extraction while, SO was the flour of the seeds discarded because they were too small or too light.

### 2.2 Chemical composition

The samples were ground with a 1‐mm screen and analysed for chemical composition (dry matter, crude protein, ether extract and ash) as reported by AOAC (2015) procedures (ID number: 2001.12, 978.04, 920.39 and 930.05 for DM, CP, EE and ash, respectively). Neutral detergent fibre, acid detergent fibre and free ash acid detergent lignin were also determined as indicated by Van Soest et al. (1991).

### 2.3 Phytocannabinoid content assessment

The phytocannabinoid contents were detected at the University of Campania, Luigi Vanvitelli (Caserta, Italy), where the substrates were first ground using mortar and pestle in liquid nitrogen, and then extracted through an ultrasound‐assisted maceration (UAM). For this purpose, an ultrasonic bath system was used (Branson UltrasonicsTM BransonicTM M3800‐E, Danbury, CT, USA), with n‐hexane as the extracting solvent. Three ultrasound cycles of 30 min each were carried out, with operating frequency set at 40 kHz, and solid to solvent ratio equal to 1:5. The obtained extracts were dried using a rotary evaporator (Heidolph Hei‐Vap Advantage, Schwabach, Germany), and reconstituted in n‐hexane for the UHPLC‐ESI‐QqTOF‐MS and MS/MS analyses. Thus, the apolar hemp by‐products extracts (10 mg/ml) were investigated using a Shimadzu NEXERA UHPLC system (Shimadzu, Tokyo, Japan) equipped with Luna Omega C18 column (50 × 2.1 mm i.d., 1.6 µm particle size). The mobile phase consisted of a binary solution composed of water (solvent A) and acetonitrile (solvent B), both acidified with formic acid (0.1% v/v). A linear gradient was used as follows: 0–1 min, 38% B; 1–5 min, 38→55% B; 5–10 min, 55% B; 10–12 min, 55→75% B; 12–14 min, 75→95% B; 14–15 min, 95% B. The total run time was 17 min with a flow rate of 0.5 mL/min; the injection volume was 2.0 µl. The AB SCIEX TripleTOF 4600 (AB Sciex, Concord, ON, Canada) system, equipped with a DuoSprayTM ion source, was combined with the UHPLC and was operated in the negative ESI mode. Data were collected by information dependent acquisition (IDA) using a TOF‐MS survey scan of 100–1,000 Da (250 ms accumulation time) and eight dependent TOF‐MS/MS scans of 80–800 Da (250 ms accumulation time). The MS parameters were as follows: curtain gas (CUR) 35 psi, nebulizer gas (GS 1) 60 psi, heated gas (GS 2) 60 psi, ion spray voltage (ISVF) 4.5 kV, interface heater temperature (TEM) 600°C and declustering potential (DP) −70 V. In TOF‐MS/MS experiments, collision energy (CE) applied was −45 kV with a collision energy spread (CES) of 25 kV. The instrument was controlled by Analyst® TF 1.7 software (AB Sciex, Concord, ON, Canada, 2016), while data processing was carried out using PeakView® software version 2.2 (AB Sciex, Concord, ON, Canada, 2016). For quantitation purposes, the calibration curves of cannabidiolic acid (CBDA) and Δ^9^‐tetrahydrocannabinolic acid (THCA), were constructed. Both the compounds were previously isolated and fully characterized by means of spectroscopic and spectrometric techniques (Formato et al., 2020). Thus, working solutions of each standard, prepared by dilution from a stock solution, were injected into the UHPLC‐ESI‐QqTOF MS system under the same conditions as the samples (Piccolella et al., 2020).

### 2.4 *In vitro* fermentation

All samples were incubated in a serum flask (seven replications per substrate ±1 g for each replication) with buffalo rumen fluid (10 ml) at 39°C under anaerobic conditions as indicated by Theodorou et al. (1994). The rumen liquor was collected, at slaughterhouse, from four buffaloes according to EU legislation (EU Council, 2004). The buffaloes fed a total mixed ration containing corn silage, oat hay and concentrate. All procedures involving animals were approved by the Ethical Animal Care and Use Committee of the University of Napoli Federico II (Prot. 2019/0013729 of 08/02/2019). The collected rumen fluids were placed inside to pre‐heated thermos and transported within 2 h to the laboratory of Feed Evaluation of University of Napoli, Federico II. The rumen fluid was mixed and strained through four layers of cheese cloths and diluted in a buffered medium (75 ml), successively, the reducing agent (4 ml) was added into the flasks (Vastolo et al., 2020). On seven replication, three bottles for each substrate were utilized for cumulative gas production measurement (120 h of incubation), the remaining were used for methane production evaluation (24 h of incubation).

The gas produced during 120 h of incubation, into the fermenting cultures, was recorded 21 times (from 2 to 24 h of intervals) using a manual pressure transducer (Cole and Palmer Instrument Co, Vernon Hills, IL, USA). The cumulative volume of gas produced after 120 h of incubation was related to incubated OM (OMCV, ml/g). At the end of the incubation period, the fermentation liquor was analysed for pH using a pH meter (ThermoOrion 720 A+, Fort Collins, CO, USA). The organic matter degradability (OMD, %) was determined by weight difference of the incubated OM and the undegraded filtered (sintered glass crucibles; Schott Duran, Mainz, Germany, porosity # 2) residue burned at 550°C for 3 h.

### 2.5 End‐products measurement

In order to determine the volatile fatty acids (VFA), the fermentation liquor was cooled at 4°C and, before analyses, centrifuged at 12,000 g for 10 min at 4°C (Universal 32R centrifuge, Hettich FurnTech Division DIY, Melle‐Neuenkirchen, Germany); the supernatant (1 ml) was then mixed with 1 ml of 0.06 mol oxalic acid. The VFA was measured by gas chromatography (ThermoQuest 8000top Italia SpA, Rodano, Milan, Italy) equipped with a fused silica capillary column (30 m, 0.25 mm ID, 0.25 μm film thickness), using an external standard solution composed of acetic, propionic, butyric, iso‐butyric, valeric and iso‐valeric acids. The percentage of branched‐chain fatty acids were calculated as: (iso‐butyric acid +iso‐valeric acid/tVFA)/100. The ammonia nitrogen (N‐NH_3_) production was assessed according to the colourimetric method proposed by Searle (1984).

### 2.6 Methane production evaluation

For each substrate, four flasks of seven were stopped at 24 h to measure the methane (CH_4_) production as described by Guglielmelli et al. (2011); the relative end‐products were also determined. The gas‐phase from each flask was sampled (3 ml) in duplicate with a gastight syringe and injected into a gas chromatograph (ThermoQuest 8000top Italia SpA, Rodano, Milan, Italy), equipped with a loop TC detector and a packed column (HaySepQ SUPELCO, 3/16‐inch, 80/100 mesh).

### 2.7 Data processing

To estimate the fermentation kinetics, for each bottle stopped at 120 h, the gas production profiles were fitted to the sigmoidal model (Groot et al., 1996):

$$G=A/{\left(1+B/t\right)}^{C}$$

where G is the total gas produced (ml per g of incubated OM) at time t (h), A is the asymptotic gas production (ml/g), B is the time at which one‐half of A is reached (h), and C is the curve switch. Maximum fermentation rate (R_max_, ml/h) and the time at which it occurs (T_max_, h) were calculated utilizing model parameters (Bauer et al., 2001):

$$R_{\text{max}}=\frac{\left(A\times C^{B}\right)\times B\times {\text{T}}_{\text{max}}^{\left(B-1\right)}}{\left(1+C^{B}\right)\times {\left({\text{T}}_{\text{max}}^{-B}\right)}^{2}}$$

$$T_{\text{max}}=C\times {\left(\frac{B-1}{B+1}\right)}^{1/B}$$

Statical analyses were performed by ANOVA for one‐way (JMP^®^, Version 14 SW, SAS Institute Inc., Cary, NC, USA, 1989–2019) to evaluate the substrate effect.

In particular, the *in vitro* parameters concerning 120 h of incubation (OMCV, OMD, T_max_, R_max_) and the data related to the end‐products (pH, VFA, BCFA, N‐NH_3_) were statically analysed, as well as the results of the methane analysis (methane production as percentage of total gas, related to incubated organic matter, and related to degraded organic matter, respectively). The significance level was verified using HSD Tukey's test at *p* < 0.01 and *p* < 0.05.

The correlations between chemical composition values and fermentation parameters and between chemical compound and methane production were also evaluated (JMP®, Version 14 SW, SAS Institute Inc., Cary, NC, USA, 1989–2019).

## 3 RESULTS

### 3.1 Chemical composition

Table 1 shows the chemical composition of the tested samples. The co‐products of hemp seeds presented a significantly higher (*p* < 0.01) level of crude protein (>30% DM) than flower co‐products. The structural carbohydrates were quite high and variable for all the tested samples. Indeed, NDF values were higher (*p* < 0.01) in seeds' samples (>38% DM) than in flowers co‐products (37 and 31% DM); lignin content was always higher than 6% DM. Moreover, both seeds co‐products showed significantly higher (*p* < 0.01) values of all structural carbohydrates' fractions. While the ether extract content of flowers co‐products was significantly (*p* < 0.05) higher than seed co‐products. Ash content of both seeds co‐products was significantly (*p* < 0.01) lower compared to flower ones.

**TABLE 1:** Chemical composition in hemp co‐products (*n* = 4)

Substrate	DM	CP	NDF	ADF	ADL	EE	Ash
	%	% DM	% DM	% DM	% DM	% DM	% DM
SF	88.64^B^	34.13^A^	39.47^BC^	28.83^A^	11.41^AB^	6.71^b^	9.16^A^
SO	91.11^A^	32.09^A^	39.86^A^	29.81^A^	11.93^A^	8.79^b^	6.98^B^
FT	88.24^B^	21.16^B^	31.29^C^	17.02^C^	6.85^C^	14.78^a^	22.91^A^
FR	88.01^B^	20.23^B^	36.85^B^	21.76^B^	7.41^BC^	17.80^a^	23.28^A^
MSE	0.12	3.44	5.65	8.74	2.51	8.01	7.50

Note

Along the column, A, B, C: *p* < 0.01; a, b: *p* < 0.05.

Abbreviations: NDF, neutral detergent fibre; ADF, acid detergent fibre; ADL, acid detergent lignin; CP, crude protein; DM, dry matter; EE, ether extract; FR, refusal of trimming of flower (rough); FT, refusal of trimming of flower (thin); SF, refusal of flour production; SO, refusal of oil extraction; MSE: mean square error.

### 3.2 Phytocannabinoid content

UHPLC‐HRMS analysis on a polar extract from the hemp co‐products matrices provided qualitative information on their phytocannabinoids' content, whereas the main acidic phytocannabinoids, namely CBDA and Δ^9^‐THCA, were quantized thanks to calibration curves available from the pure isolated reference compounds (Piccolella et al., 2020). CBDA and Δ^9^‐THCA metabolites showed the deprotonated molecular ion ([M‐H]^−^) ion at *m/z* 357.21 according to the molecular formula C_22_H_30_O_5_. The constitutional isomers were identified based on their TOF‐MS/MS fragmentation pattern and their relative retention time (Formato et al., 2020; Piccolella et al., 2020). In particular, FR and FT extracts contained a greater amount of the two compounds with respect to SO and SF samples (Table 2).

**TABLE 2:** Cannabidiolic and Δ^9^‐Tetrahydrocannabinolic acids amount (average ± SD) in hemp co‐products extract (*n* = 4)

Extract	Compounds	
	CBDA	Δ^9^‐THCA
	mg/g	mg/g
SF	2.80 ± 0.21	0.011 ± 0.0003
SO	4.45 ± 0.76	0.089 ± 0.004
FT	19.00 ± 1.7	1.850 ± 0.090
FR	16.50 ± 1.3	1.250 ± 0.050

Abbreviations: FR, refusal of trimming of flower (rough); FT, refusal of trimming of flower (thin); SF, refusal of flour production; SO, refusal of oil extraction; THCA, Δ^9^‐Tetrahydrocannabinolic acid; CBDA, Cannabidiolic acid.

This could be due to the plant part origin, which was the aerial part for both FR and FT samples, and the fruit (also known as hemp seed) for SO and SF samples. The lowest content of both phytocannabinoid was detected in the flours obtained after the oil extraction from the hemp seed. However, FR and FT extracts showed a high diversity in phytocannabinoids. Indeed, it was observed that FR and FT extracts contained, beyond CBDA and Δ^9^‐THCA, other acidic phytocannabinoids such as CBDA‐C_4_ and cannabivarinic acid with respective [M‐H]^−^ ion at *m/z* 343.1906 and 329.1755. Moreover, cannabifuranic acid (CBFA), cannabinolic acid (CBNA) and cannabinodiolic acid (CBNDA), which share the [M‐H]^−^ ion at *m/z* 353.17 (C_22_H_26_O_4_), were also identified. Furthermore, TOF‐MS experiment detected eleven compounds with the [M‐H]^−^ ion at *m/z* 373.20 (C_22_H_30_O_5_), among which TOF‐MS/MS data allowed us to recognize cannabielsoic acids (CBEA‐C_5_ A and B) and epoxy‐phytocannabinoid acids. Considering the relative areas, these metabolites represented the 57.94% in FR and 49.28% in FT. This latter sample also contained cannabigerolic acid (CBGA), 6,7‐epoxycannabigerolic acid (C_22_H_32_O_5_) and its neutral form, 6,7‐epoxycannabigerol (C_21_H_32_O_3_) in a relative percentage equal to 2.8%. Finally, five metabolites with [M‐H]^−^ ion at *m/z* 389.19, which could be O_6_‐type phytocannabinoids, were three‐fold higher in FR than in FT extract.

### 3.3 *In vitro* fermentation characteristics

**FIGURE 1:**
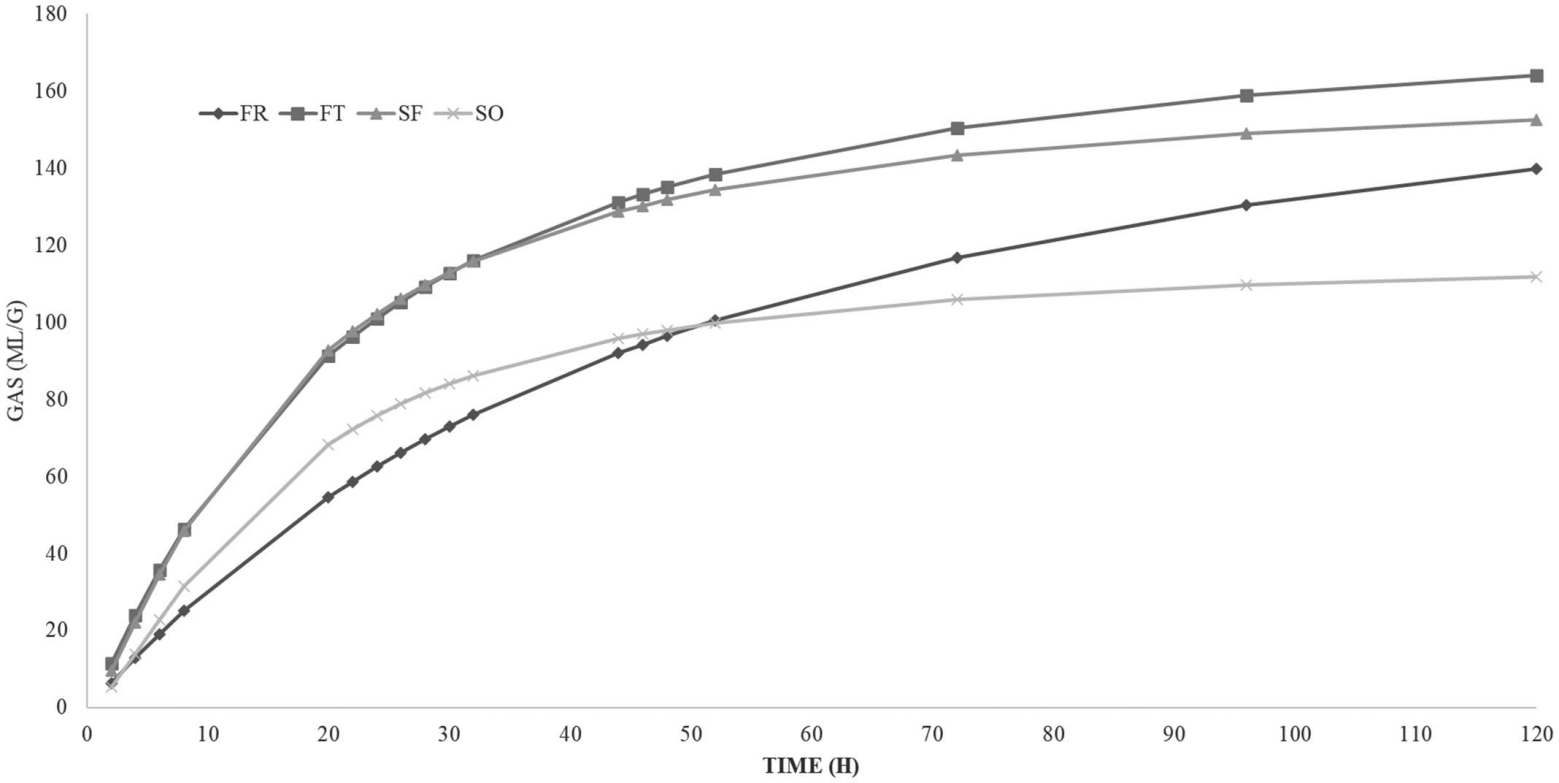
*In vitro* gas production over time in hemp co‐products

**FIGURE 2:**
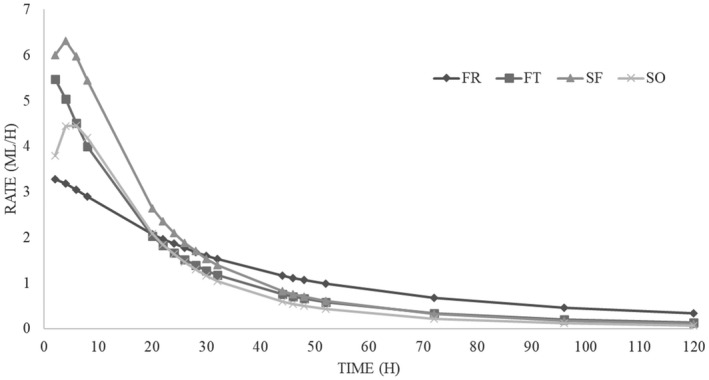
*In vitro* fermentation rate in hemp co‐products

The *in vitro* fermentation characteristics and kinetics are reported in Table 3. The refusal of flour production (SF) showed OMD values significant higher (*p* < 0.05) compared to FR. The cumulative gas production parameter showed highly significant differences between substrates (*p* < 0.01). Thin refusal of trimming flower (FT) resulted in the highest (*p* < 0.01) OMCV values and SO the lowest. Regarding the kinetic parameters (T_max_ and R_max_), the *in vitro* fermentation rate of all hemp co‐products was lower than 7 ml/h and the maximum rate was reached at the beginning of the incubation, within 5 h. In particular, FT presented the highest fermentation rate (*p* < 0.01) compared to SO and FR. Whilst SO showed a significantly high (*p* < 0.01) T_max_ values compared to the other substrates. All these findings were better represented in Figures 1 and 2.

**TABLE 3:** *In vitro* fermentation characteristics in hemp co‐products (*n* = 3)

Substrate	OMD %	OMCV ml/g	R_max_ ml/h	T_max_ h
SF	45.65^a^	149.91^B^	6.33^A^	3.60^B^
SO	35.63^ab^	110.11^C^	4.66^B^	4.95^A^
FT	30.08^b^	164.13^A^	4.86^AB^	3.00^B^
FR	28.09^b^	136.37^B^	3.21^C^	2.35^B^
MSE	4.97	6.24	0.54	0.52

Note

Along the column, different capital superscript letters indicate difference for *p* < 0.01; different lowercase superscript letters indicate difference for *p* < 0.05. MSE: mean square error.

Abbreviations: SF, refusal of flour production; SO, refusal of oil extraction; FR, refusal of trimming of flower (rough); FT, refusal of trimming of flower (thin); OMD, organic matter degradability; OMVC, cumulative volume of gas related to incubate organic matter; R_max_, maximum fermentation rate; T_max_, time at which R_max_ occurs.

### 3.4 **End‐products parameters after 120** **h of incubation**

In Table 4 the *in vitro* end‐products parameters are reported. No statically differences between the tested substrates for pH values emerged. Volatile fatty acid production differs between substrates. Particularly, SO showed the lowest tVFA value (75.58 Mmol/g OM; *p* < 0.01) and highest values of BCFA (9.60% VFA; *p* < 0.01) and N‐NH_3_ (62.69 mg/g OM; *p* < 0.01). Acetate and propionate were the most representative VFA of all the substrates; they represented 80% of the total. SO showed the lowest proportion of acetate (56.71% tVFA) while SF had the lowest proportion of propionate (18.47% tVFA). The flower co‐products showed significantly higher (*p* < 0.01) acetate and propionate production compared to seeds co‐products. The ratio acetate/propionate was significantly higher for SF compared to the other samples. Regarding butyrate production, the seeds co‐products showed the highest production compared to flowers co‐products.

**TABLE 4:** *In vitro* fermentation end products evaluated after 120 h of incubation (*n* = 6)

Substrate	pH	tVFA	Ace	Prop	Iso‐But	But	Iso‐val	Val	BCFA	Ace/Prop	N‐NH_3_
		Mmol/gOM	%tVFA	%VFA	%VFA	%VFA	%VFA	%VFA	%VFA		mg/gOM
SF	6.47	92.05^A^	67.36^A^	18.47^B^	1.86^B^	7.12^AB^	3.45^B^	1.74^B^	5.31^B^	3.64^A^	54.4^B^
SO	6.44	75.58^B^	56.71^B^	22.59^A^	3.49^A^	7.27^A^	6.16^A^	3.82^A^	9.60^A^	2.56^B^	62.69^A^
FR	6.44	99.60^A^	64.27^A^	23.57^A^	1.72^B^	6.15^BC^	2.62^BC^	1.81^B^	3.42^C^	2.75^B^	50.55^B^
FT	6.47	98.04^A^	65.16^A^	24.65^A^	1.19^C^	5.31^BC^	2.21^C^	1.22^C^	5.12^B^	2.64^B^	51.97^B^
MSE	0.002	36.47	3.52	5.04	0.08	0.51	0.28	0.05	0.0027	0.10	3.88

Note

Along the column, different capital letters indicate difference for *p* < 0.1.

Abbreviations: SF, refusal of flour production; SO, refusal of oil extraction; tVFA, total volatile fatty acids; Ace, acetate; Ace/Prop, ratio between acetate and propionate; FR, refusal of trimming of flower (rough); Prop, propionate; Iso‐But, Isobutyrate; But, butyrate; Isoval, Isovalerate; BCFA, branched‐chain fatty acids; FT, refusal of trimming of flower (thin); N‐NH_3_, ammonia; tVFA, total volatile fatty acids; Val, valerate; MSE, mean square error.

### 3.5 Methane production

The methane production registered after 24 h of incubation, the organic matter degradability and relative tVFA values are reported in Table 5. The refusal of flour production resulted in the highest (*p* < 0.01) CH_4_ values when reported as a percentage of total gas and related to incubated organic matter. If methane production was related to OMD, FT and SF showed significantly higher methane production. On contrary, FR showed the lowest (*p* < 0.01) CH_4_ production to all parameters. After 24 h of incubation, FR and SF showed the highest (*p* < 0.01) levels of OMD, while FT and SF showed the highest (*p* < 0.01) levels of total VFA. The refusal of oil extraction showed the lowest (*p* < 0.01) value for both parameters.

**TABLE 5:** *In vitro* methane production and main volatile fatty acids values obtained after 24 h incubation (*n* = 4)

Substrate	pCH_4_	iCH_4_	dCH_4_	OMD_24_ _h_	tVFA_24_ _h_
	%Total gas	ml/g iOM	ml/g OMD_24_ _h_	%	Mmol/g OM
SF	8.24^A^	3.88^A^	10.39^A^	37.7^A^	40.47^AB^
SO	6.43^AB^	1.34^C^	5.80^B^	23.22^B^	34.87^B^
FT	5.56^B^	2.00^B^	11.73^A^	27.18^AB^	53.24^A^
FR	4.80^B^	0.56^D^	1.78^B^	31.46^A^	38.76^B^
MSE	0.11	0.08	0.75	3.32	5.20

Note

Along the column, different capital letters indicate difference for *p* < 0.01.

SF, refusal of flour production; SO, refusal of oil extraction; FT, refusal of trimming of flower (thin); FR, refusal of trimming of flower (rough); pCH4, methane production as percentage of total gas; OMD24 h, organic matter degradability after 24 h of incubation; dCH_4_, methane production related to degraded organic matter; iCH_4_, methane production related to incubated organic matter; tVFA_24_ _h_, total volatile fatty acids after 24 h of incubation; MSE, mean square error.

## 4 DISCUSSION

Regarding chemical composition, the data of hemp co‐products of this study agreed to that reported in literature (Alaru et al., 2011; Gibb et al., 2005; Vonapartis et al., 2015) for flower and seeds co‐products. In this regard, hemp co‐products chemical composition, particularly of seeds, could be comparable to soybean meal ones (Semwogerere et al., 2020). Indeed, taking into count the high protein content and considering the high amount of essential amino acids reported by House et al. (2010) for similar substrates, all tested co‐products could be evaluated as a source of high‐quality protein. Moreover, the crude protein amount of hemp co‐products is higher than the endorsed ruminant dietary requirement for maintenance and growth (CSIRO, 2007). In particular, these co‐products seem to be able to satisfy the nutritional requirements for buffaloes in early lactation as indicating by Bartocci et al. (2002). As reported by Mustafa et al. (1999) the hemp oil meal obtained by mechanical oil extraction could be considered excellent natural sources of rumen undegradable protein. These authors included hemp oil meal in the diet for sheep (20% DM) and did not observe a detrimental effect. No data in the literature were present about protein degradability into the rumen of hemp discarded seeds and flower co‐products. In particular, these last showed interesting protein and NDF contents comparable to legume forages usually utilized in ruminant nutrition such as berseem clover (Sabia et al., 2015; Tsiobani et al., 2019). The high content of structural carbohydrates could make available a high energy level in these substrates. Nevertheless, the particularly high amount of lignin could limit the availability of these resources (House et al., 2010). However, the active molecules present in the hemp might cause an overestimation of lignin, as suggested by Marles et al. (2008) for substrates rich in condensed tannins. Similarly, the ether extract values could be influenced by the high content of resins in hemp flowers (Formato et al., 2020). Both flowers' co‐products showed higher amounts of acidic phytocannabinoids. CBDA and THCA were the most abundant, whereas some other derivatives with higher oxygenation degrees or with different alkyl chains were detected thanks to the sensitivity of high‐resolution spectrometric tools. These results are according to the observation of Kleinhenz et al. (2020) which detected six different cannabinoids in a different portion of the hemp plant such as stalks, flowers, leaves and seeds. These authors indicated that phytocannabinoids were present in all plant fractions and that CBDA and THCA were always the most concentrated. It is fair to mention that the determination of the qualitative profile in phytocannabinoids and the quantization of the individual substances in the raw materials selected for feed (panel‐cake, oil, flour and hemp fibre) is mandatory according to EU Reg. 1017/2017). The compositional analyses of cannabinoid levels will be useful to define the admissibility of raw materials based on hemp for animal feed, since the compliance of the raw material, already defined in Reg. (EC) 767/09, takes into account a maximum content of Δ^9^‐THC equal to 0.2%. The ash amount registered in the study for all samples was higher compared to the literature data (Callaway, 2004; Semwogerere et al., 2020) in particular for FR and FT. A lot of factors such as botanical species, type of soil, fertilization, harvesting and processing methods could affect the mineral profile of samples (McDonald et al., 2011).

Regarding the *in vitro* gas production, unlike the chemical composition, there are no more studies about hemp (*Cannabis sativa* L.) plants and/or co‐products. Only Kleinhenz et al. (2020) studied the *in vitro* degradability of different portions of the hemp plant (whole plants, stalks, unprocessed female flowers, whole seed heads, dried leaves, chaff and processed female flowers). They concluded that seed heads, chaff, and leaves had the lowest undegradable NDF amount after 240 h of incubation and observed a percentage of degradability similar to that registered in this study. In particular, the higher acetate and propionate production registered in rumen fermentation liquor of flower co‐products could indicate that the structural carbohydrates of these substrates were better fermented compared to seeds co‐products ones. Concerning of the kinetics of fermentation, all substrates showed the maximum fermentation rate (ml/h) in the first 5 h of incubation and after it decreased rapidly within 20 h of incubation. The higher concentration of fat of both flower co‐products could have affected negatively their fermentation processes.

The substrates SO and SF showed the highest OMD and the lowest OMCV values. The most intense fermentation could be due to the high crude protein concentration probably, considering that protein fermentation did not produce gas (Abreu and Bruno Soares, 1998). When the microorganisms are not able to ferment the carbohydrates, they have to use other nutrients such as protein, in order to survive. Also the highest BCFA proportion an N‐NH_3_ production registered by these substrates after 120 h of incubation could indicate a protein fermentation. Moreover, the higher values of BCFA and ammonia were significantly related to the protein content (BCFA vs. CP: *r* = 0.813, *p* < 0.05 and N‐NH_3_ vs. CP: *r* = 0.727, *p* < 0.05); indeed, no correlation was found between BCFA and N‐NH_3_ production, as described by Davila et al. (2013) this could be due to the specific amino acids profile. In this regard, Isobutyrate and Isovalerate production are the result of branched‐chains amino acids metabolism, such as valine, leucine and isoleucine; they can be hydrolyzed and fermented to phenols, and biogenic amines (i.e., indole, skatole, 4‐ethylphenol, p‐cresol) (Musco et al., 2018). Semwogerere et al. (2020) reported the amino acid profile of seeds and different co‐products indicating that leucine and valine were among the main represented amino acids in hemp.

The shape representing the fermentation rate of the hemp co‐products indicates that FR is a quite slow substrate, probably due to some component that does not facilitate the enzymatic attack by microorganisms. On the contrary, FT and SF show a rapid fermentation process, due to an easily fermentable component. About the SO, the curve is in an intermediate position.

Regarding the methane production, the data of this study seem particularly low compared to that one obtained in previous studies (Calabrò et al., 2012; Guglielmelli et al., 2011) and reported by other authors (Tuyen et al., 2013; Elghandour et al., 2016), probably due to the different experimental condition in terms of hours of incubation. The limited methane production could be ascribed to specific nutritional characteristics of hemp co‐products or to the phytocannabinoids presence. The negative relation (EE vs. CH_4_: *r* = −8792; *p* < 0.05) between ether extract content and CH_4_ (% total gas) production observed in this study, suggest an inhibiting role of the lipid fraction on methanogenesis. In literature, only a few studies are referred to these substrates. Particularly, Wang et al. (2017) measured the *in vitro* methane production of diets characterized by different lipids sources (seeds of safflower, poppy, hemp and camelina vs. coconut and linseed). These authors demonstrated that the use of sunflower and hemp seeds was more efficient than linseed in abating the level of methanogenesis. They suggested that the results were not affected by ω6/ω3 fatty acids or C18:2/C18:3 ratios. Moreover, they suggest a possible role of uncommon fatty acids (C18:3, ω6 and C18:4, ω3) present in hemp and camelina seeds on methanogens microbes. Shibata and Terada (2010) indicated that the supplementation of unsaturated fatty acids (linoleic, α‐linolenic and oleic acids) in the ruminant's diet inhibits CH_4_ production into the rumen.

A significant correlation between OMCV and both phytocannabinoids concentrations (CBDA vs. OMCV, *r* = 0.548; *p* < 0.01; TCHA vs. OMCV, *r* = 0.617; *p* < 0.0001) could indicate a positive interference between *in vitro* fermentation and these compounds. No correlations were observed between OMD and cannabinoids or methane production and cannabinoids. All these results suggest that acidic phytocannabinoids, mainly CBDA and TCHA could affect the fermentation pathways without limiting the organic matter degradability.

## 5 CONCLUSION

These preliminary results underline how the use of residues of hemp processing could be a valid nutrient resource in the diet of ruminants. Indeed, hemp co‐products showed interesting nutritive values as demonstrated by the results of the chemical composition. However, *in vitro* data (i.e. OMD and OMCV) indicate as these substrates are quite low utilized by the rumen microbes. However, the low methane values suggest as these samples could be used in the ruminant diets to contain gas emission, and therefore, the environmental impact. Further studies are needed to evaluate if it is possible to improve the hemp structural carbohydrates utilization fermentability and to investigate the mechanisms that determine the trend registered *in vitro*. Moreover, could be interesting to conduce *in vivo* studies to understand in what doses these co‐products could be included in ruminants' diet and if they could be useful to mitigate the environmental impact of intensive farm.

## CONFLICT OF INTEREST

The authors declare that they have no competing interests.

## AUTHORS' CONTRIBUTIONS

SC and MIC designed the research. AV collected the data. AV, BIK and SP made the laboratory analysis. BIK and AV analysed the data. BIK and AV wrote the manuscript. SC, SP and MIC reviewed and edited the manuscript. All the authors read and approved the final manuscript.

## ANIMAL WELFARE STATEMENT

All procedures involved animals were approved by the Ethical Animal Care and Use Committee of the University of Napoli Federico II (Prot. 2019/0013729 of 08/02/2019).

## Data Availability

The data sets used and/or analysed during the current study are available from the corresponding author on reasonable request.

## References

[jpn13557-bib-0001] Abreu, J. M. F. , & Bruno‐Soares, A. M. (1998). Chemical composition, organic matter digestibility and gas production of nine legume grains. Animal Feed Science and Technology, 70, 49–57. 10.1016/S0377-8401(97)00071-0

[jpn13557-bib-0002] Alaru, M. , Kukk, L. , Olt, J. , Menind, A. , Lauk, R. , Vollmera, E. , & Astoverc, A. (2011). Lignin content and briquette quality of different fibre hemp plant types and energy sunflower. Fields Crops Research, 124, 332–339.

[jpn13557-bib-0003] AOAC (2015). Official Methods of Analysis (20th ed.). Association of Official Analytical Chemists.

[jpn13557-bib-0004] Bartocci, S. , Tripaldi, C. , & Terramoccia, S. (2002). Characteristics of foodstuffs and diets, and the quanti‐qualitative milk parameters of Mediterranean buffaloes bred in Italy using the intensive system An estimate of the nutritional requirements of buffalo herds lactating or dry. Livestock Production Science, 77, 45–48. 10.1016/S0301-6226(02)00022-2

[jpn13557-bib-0005] Bauer, E. , Williams, B. A. , Voigt, C. , Mosenthin, R. , & Verstegen, M. W. A. (2001). Microbial activities of faeces from unweaned and adult pigs, in relation to selected fermentable carbohydrates. Journal of Animal Science, 73, 313–322.

[jpn13557-bib-0006] Calabrò, S. , Guglielmelli, A. , Iannaccone, F. , Danieli, P. P. , Tudisco, R. , Ruggiero, C. , Piccolo, G. , Cutrignelli, M. I. , & Infascelli, F. (2012). Fermentation kinetics of sainfoin hay with and without PEG. Journal of Animal Physiology and Animal Nutrition, 96, 842–849. 10.1111/j.1439-0396.2011.01260.x 22168179

[jpn13557-bib-0007] Callaway, J. C. (2004). Hempseed as a nutritional resource: An overview. Euphytica, 104, 65–72.

[jpn13557-bib-0008] EC Council (2004). Regulation 882/2004 on Official controls performed to ensure verification of compliance with feed and food law, animal health and animal welfare rules. Official Journal of European Union, L191/1, 1–52.

[jpn13557-bib-0009] Crini, G. , Lichtfouse, E. , Chanet, G. , & Morin‐Crini, N. (2020). Applications of hemp in textiles, paper industry, insulation and building materials, horticulture, animal nutrition, food and beverages, nutraceuticals, cosmetics and hygiene, medicine, agrochemistry, energy production and environment: a review. Environmental Chemistry Letters, 18, 1451–1476. 10.1007/s10311-020-01029-2

[jpn13557-bib-0010] CSIRO (2007). Nutrient requirements of domesticated ruminants. In M. Freer , H. Dove , & J. V. Nolan (Eds.), Nutrient Requirements of Domesticated Ruminants. CSIRO Pub.

[jpn13557-bib-0011] Davila, A. M. , Blachier, F. , Gotteland, M. , Andriamihaja, M. , Benetti, P. H. , Sanz, Y. , & Tomé, D. (2013). Intestinal luminal nitrogen metabolism: Role of the gut microbiota and consequences for the host. Pharmacological Research, 68, 95–107. 10.1016/j.phrs.2012.11.005 23183532

[jpn13557-bib-0012] Elghandour, M. M. Y. , Kholif, A. E. , Salem, A. Z. M. , Olafadehan, O. A. , & Kholif, A. M. (2016). Sustainable anaerobic rumen methane and carbon dioxide productions from prickly pear cactus flour by organic acid salts addition. Journal of Cleaner Production, 139, 1362–1369. 10.1016/j.jclepro.2016.08.075

[jpn13557-bib-0013] FAOSTAT (2020). Retrieved from: http://www.fao.org/faostat/en/#data.

[jpn13557-bib-0014] Farinon, B. , Molinari, R. , Costantini, L. , & Merendino, N. (2020). The Seed of Industrial Hemp (Cannabis sativa L.): Nutritional Quality and Potential Functionality for Human Health and Nutrition. Nutrients, 12, 1935. 10.3390/nu12071935 PMC7400098 32610691

[jpn13557-bib-0015] Faugno, S. , Piccolella, S. , Sannino, M. , Principio, L. , Crescente, G. , Baldi, G. M. , Fiorentino, N. , & Pacifico, S. (2019). Can agronomic practices and cold‐pressing extraction parameters affect phenols and polyphenols content in hempseed oils? Industrial Crop and Products, 130, 511–519. 10.1016/j.indcrop.2018.12.084

[jpn13557-bib-0016] Fiorini, D. , Molle, A. , Nabissi, M. , Santini, G. , Benelli, G. , & Maggi, F. (2019). Valorizing industrial hemp (Cannabis sativa L.) by‐products: Cannabidiol enrichment in the inflorescence essential oil optimizing sample pre‐treatment prior to distillation. Industrial Crops & Products, 128, 581–589. 10.1016/j.indcrop.2018.10.045

[jpn13557-bib-0017] Formato, M. , Crescente, G. , Scognamiglio, M. , Fiorentino, A. , Pecoraro, M. T. , Piccolella, S. , Catauro, M. , & Pacifico, S. (2020). (‐)‐Cannabidiolic acid, a still overlooked bioactive compound: An introductory review and preliminary research. Molecules, 25, 2638. 10.3390/molecules25112638 PMC7321064 32517131

[jpn13557-bib-0018] Gibb, D. J. , Shah, M. A. , Mir, P. S. , & McAllister, T. A. (2005). Effect of full‐fat hemp seed on performance and tissue fatty acids of feedlot cattle. Canadian Journal of Animal Science, 85, 223–230.

[jpn13557-bib-0019] Groot, J. C. J. , Cone, J. W. , Williams, B. A. , Debersaques, F. M. A. , & Lantinga, E. A. (1996). Multiphasic analysis of gas production kinetics for in vitro fermentation of ruminant feedstuff. Journal of Feed Science and Technology, 64, 77–89.

[jpn13557-bib-0020] Guglielmelli, A. , Calabrò, S. , Primi, R. , Carone, F. , Cutrignelli, M. I. , Tudisco, R. , Piccolo, G. , Ronchi, B. , & Danieli, P. P. (2011). In vitro fermentation patterns and methane production of sainfoin (Onobrychis viciifolia Scop.) hay with different condensed tannin contents. Grass and Forage Science, 66, 488–500. 10.1111/j.1365-2494.2011.00805

[jpn13557-bib-0021] House, J. D. , Neufeld, J. , & Leson, G. (2010). Evaluating the quality of protein from hemp seed (Cannabis sativa L.) products through the use of the protein digestibility‐corrected amino acid score method. Journal of Agriculture and Food Chemistry, 58, 11801–11807. 10.1021/jf102636b 20977230

[jpn13557-bib-0022] Kleinhenz, M. D. , Magnin, G. , Ensley, G. S. , Griffin, J. J. , Goeser, J. , Lynch, E. , & Coetzee, J. F. (2020). Nutrient concentrations, digestibility, and cannabinoid concentrations of industrial hemp plant components. Applied Animal Science, 36, 489–494.

[jpn13557-bib-0023] Marles, S. , Bruce, M. A. , Coulman, E. , & Bett, K. E. (2008). Interference of condensed tannin in lignin analyses of dry bean and forage crops. Journal of Agriculture and Food Chemistry, 56, 9797–9802. 10.1021/jf800888r 18841900

[jpn13557-bib-0024] McDonald, P. , Edwards, R. A. , Greenhalgh, J. F. D. , Morgan, C. A. , Sinclair, L. A. , & Wilkinson, R. G. (2011). Animal Nutrition (7th ed.). Pearson Education Limited.

[jpn13557-bib-0025] Milanovic, J. , Kostić, M. , Milanovic, P. , & Skundric, P. (2012). Influence of TEMPO‐mediated oxidation on properties of hemp fibres. Industrial & Engineering Chemistry Research, 51, 9750–9759. 10.1021/ie300713x

[jpn13557-bib-0026] Musco, N. , Calabrò, S. , Roberti, F. , Grazioli, R. , Tudisco, R. , Lombardi, P. , & Cutrignelli, M. I. (2018). In vitro evaluation of Saccharomyces cerevisiae cell wall fermentability using a dog model. Journal of Animal Physiology and Animal Nutrition, 102(1), 24–30. 10.1111/jpn.12864 29623689

[jpn13557-bib-0027] Mustafa, F. , McKinnon, J. J. , & Christensen, D. A. (1999). The nutritive value of hemp meal for ruminants. Canadian Journal of Animal Science, 79, 92–95. 10.4141/A98-031

[jpn13557-bib-0028] Piccolella, S. , Crescente, G. , Formato, M. , & Pacifico, S. (2020). A cup of hemp coffee by moka pot from Southern Italy: An UHPLC‐HRMS investigation. Foods, 9(8), 1123. 10.3390/foods9081123 PMC7466224 32824076

[jpn13557-bib-0029] Ranelli, P. , & Venturi, G. (2004). Hemp as a raw material for industrial applications. Euphytica, 140, 1–6.

[jpn13557-bib-0030] Sabia, E. , Claps, S. , Napolitano, F. , Annicchiarico, G. , Bruno, A. , Francaviglia, R. , Sepe, L. , & Aleandri, R. (2015). In vivo digestibility of two different forage species inoculatedwith arbuscular mycorrhiza in Mediterranean red goats. Small Ruminant Research, 123, 83–87. 10.1016/j.smallrumres.2014.10.008

[jpn13557-bib-0031] Searle, P. L. (1984). The Berthelot or indophenol reaction and its use in the analytical chemistry of nitrogen. A Review. Analyst, 109, 549–568. 10.1039/an9840900549

[jpn13557-bib-0032] Semwogerere, F. , Katiyatiya, C. L. F. , Chikwanha, O. C. , Marufu, M. C. , & Mapiye, C. (2020). Bioavailability and bioefficacy of hemp by‐products in ruminant meat production and preservation: A review. Frontiers in Veterinary Science, 7, 572906. 10.3389/fvets.2020.572906 33102571 PMC7545362

[jpn13557-bib-0033] Shibata, M. , & Terada, F. (2010). Factors affecting methane production and mitigation in ruminants. Animal Science Journal, 81, 2–10. 10.1111/j.1740-0929.2009.00687.x 20163666

[jpn13557-bib-0034] Theodorou, M. K. , Williams, B. A. , Dhanoa, M. S. , McAllan, A. B. , & France, J. A. (1994). Simple gas production method using a pressure transducer to determine the fermentation kinetics of ruminant feeds. Animal Feed Science and Technology, 48, 185–197.

[jpn13557-bib-0035] Tsiobani, E. T. , Yiakoulaki, M. D. , & Menexes, G. (2019). Seasonal variation in water buffaloes' diet grazing in wet grasslands in Northern Greece. Hacquetia, 18(2), 201–2012. 10.2478/hacq-2019-0004

[jpn13557-bib-0036] Tuyen, D. V. , Phuong, H. N. , Cone, J. W. , Baars, J. J. P. , Sonnenberg, A. S. M. , & Hendriks, W. H. (2013). Effect of fungal treatments of fibrous agricultural by‐products on chemical composition and in vitro rumen fermentation and methane production. Bioresource Technology, 129, 256–263. 10.1016/j.biortech.2012.10.128 23261998

[jpn13557-bib-0037] Van Soest, P. J. , Robertson, J. B. , & Lewis, B. A. (1991). Methods for dietary fiber, neutral detergent fiber, and non‐starch polysaccharides in relation to animal nutrition. Journal of Dairy Science, 74, 3583–3597. 10.3168/jds.S0022-0302(91)78551-2 1660498

[jpn13557-bib-0038] Vastolo, A. , Calabrò, S. , Cutrignelli, M. I. , Raso, G. , & Todaro, M. (2020). Silage of Prickly Pears (Opuntia spp.) juice by‐products. Animals, 10, 1716. 10.3390/ani10091716 PMC7552651 32971897

[jpn13557-bib-0039] Vonapartis, E. , Aubin, M. P. , Seguin, P. , Mustafa, A. F. , & Charron, J. B. (2015). Seed composition of ten industrial hemp cultivars approved for production in Canada. Journal of Food Composition and Analysis, 39, 8–12. 10.1016/j.jfca.2014.11.004

[jpn13557-bib-0040] Wang, S. , Kreuzer, M. , Braun, U. , & Schwarm, A. (2017). Effect of unconventional oilseeds (safflower, poppy, hemp, camelina) on in vitro ruminal methane production and fermentation. Journal of Science Food and Agriculture, 97, 3864–3870. 10.1002/jsfa.8260 28188639

[jpn13557-bib-0041] Żuk‐Gołaszewska, K. , & Gołaszewski, J. (2018). Cannabis sativa L. Cultivation and quality or raw material. Journal of Elementology, 23, 971–984. 10.5601/jelem.2017.22.3.1500

